# Data on work-related consequences of COVID-19 pandemic for employees across Europe

**DOI:** 10.1016/j.dib.2020.106174

**Published:** 2020-08-18

**Authors:** Jakub Prochazka, Tabea Scheel, Petr Pirozek, Tomas Kratochvil, Cristina Civilotti, Martina Bollo, Daniela Acquadro Maran

**Affiliations:** a Masaryk University, Department of Corporate Economy, Lipova 41a, Brno 60200, Czech Republic; b Europa-Universitaet Flensburg, Germany; c Università degli Studi di Torino, Italy

**Keywords:** COVID-19, Lockdown, Job attitudes, Work performance, Coping, Resilience

## Abstract

The COVID-19 pandemic influenced the work of employees across all continents. This article presents raw data that may be used to describe how the pandemic affected the work of employees in four European countries and how it influenced their job attitudes, feelings and work performance. In total, 726 respondents from Germany, the Czech Republic, Slovakia and Italy filled out an extensive online survey and provided information about changes in their workload, work difficulty, income, social contact, work from home, task performance and organizational commitment during the pandemic, and about the risk of being infected by COVID-19 during their workday. The employees also reported their actual work performance, organizational commitment, job satisfaction, intention to leave and irritation in the time of the pandemic. To reveal factors that might help employees cope with pandemic, the respondents filled out established questionnaires measuring servant leadership of their supervisor, perceived organizational support, social support provided by colleagues, their own occupational self-efficacy, resilience, job crafting and readiness for change. The data is unique as it was collected in a specific situation during the pandemic, when the work of employees was affected by security measures and lockdown introduced by governments in countries where they worked.

**Specifications Table**

**Table:** 

**Subject**	Organizational Behavior and Human Resource Management
**Specific subject area**	Job attitudes, work performance, coping, well-being
**Type of data**	Raw data (.sav, .csv, .xls), tables with descriptive statistics
**How data were acquired**	Data were gathered through an online survey in 4 countries.
**Data format**	Raw, descriptive statistics
**Parameters for data collection**	Respondents were adults who were employed in Germany, the Czech Republic, Slovakia and Italy during the COVID-19 pandemic and worked in their organization for at least 5 months.
**Description of data collection**	The data were collected in May 2020 in Germany, the Czech Republic and Slovakia, and in May 2020 and June 2020 (until 4th of June) in Italy. The respondents participated in an online survey.
**Data source location**	Country: Germany, Czech Republic, Slovakia, Italy
**Data accessibility**	Repository name: Mendeley Data Data identification number: 10.17632/77dcsp2vcw.2 Direct URL to data: http://dx.doi.org/10.17632/77dcsp2vcw.2

**Value of the Data**

• Dataset enables analysing the work-related impact of COVID-19 pandemic on employees in 4 European countries.
• Researchers can use the dataset to analyze how employees perceived their work and their organization during the pandemic and which personal, organizational and socio-demographic factors helped employees cope with the pandemic.
• Researchers can use the dataset to test models of coping with extraordinary situations.
• The data is unique as it was collected in a specific situation during a pandemic, when the work of employees was affected by security measures and lockdown.
• The data is unique as it provides information about 15 important constructs from the area of human resource management and work psychology that were measured by established questionnaires in 4 different countries.

## 1 Data description

The COVID-19 pandemic plunged Europe into a crisis in the first half of 2020. To reduce the spread of the virus, governments have introduced lockdown and security measures. In the Czech Republic, the government declared a state of emergency on March 12th and introduced a number of new restrictions between March 14th and 30th (Ministry of Health of the Czech Republic, https://koronavirus.mzcr.cz/vyvoj-udalosti-v-case/). From April 14th, a plan for the gradual release of security measures was announced. The state of emergency was lifted on May 17th, but a number of measures lasted until the end of June or even longer (Government of the Czech Republic, https://www.vlada.cz/cz/epidemie-koronaviru/dulezite-informace/mimoradna-opatreni-_-co-aktualne-plati-180234/).

In Slovakia, the government declared a state of emergency on March 11th and lifted it on June 14th. During the first weeks of the state of emergency, the government introduced several restrictions. Starting on April 22nd, the first phase of the gradual release of security measures has begun. The eighth phase of release started on July 1st and even after this date some restrictions still applied (Government office of the Slovak Republic, https://korona.gov.sk).

**Table 1:** Characteristics of the sample (interval variables).

		Age	Number of children	Number of employers during career	Years in current organization	Workload (hours per week)
Germany	Valid *N*	135	136	137	137	137
	Missing	12	11	10	10	10
	*M*	40.47	1.01	4.19	8.33	33.04
	*SD*	12.11	1.36	2.48	8.85	10.63
	Med	41.00	0.00	4.00	4.00	38.00
	Min	19	0	1.00	0.50	3
	Max	63	7	16	37	60
Czech Republic	Valid *N*	232	230	231	229	229
	Missing	21	23	22	24	24
	*M*	32.69	.63	3.35	7.28	37.03
	*SD*	9.20	.99	2.10	31.66	11.03
	Med	29.00	.00	3.00	3.00	40.00
	Min	20	0	1.00	.50	0
	Max	65	4	16	475	80
Slovakia	Valid *N*	164	164	164	163	161
	Missing	3	3	3	4	6
	*M*	41.62	1.17	3.59	10.06	39.57
	*SD*	11.22	1.85	2.04	9.28	10.37
	Med	41.00	1.00	3.00	7.00	40.00
	Min	23	0	1.00	.50	8
	Max	71	20	10	47	96
Italy	Valid *N*	151	151	150	150	149
	Missing	8	8	9	9	10
	*M*	48.76	1.21	3.93	16.91	34.68
	*SD*	11.19	0.91	2.33	12.91	7.39
	Med	52.00	1.00	3.00	15.00	36.00
	Min	21	0	1.00	.60	7
	Max	65	3	14	41	55

**Table 2:** Characteristics of the sample (nominal variables).

		Germany		Czech Rep.		Slovakia		Italy	
		Freq.	%	Freq.	%	Freq.	%	Freq.	%
	Total	147	20.2	253	34.8	167	23.0	159	21.9
Country of origin	Germany	133	90.5	0	0.0	0	.0	0	.0
	Czech Republic	0	.0	203	80.2	7	4.2	0	.0
	Slovakia	4	2.7	43	17.0	158	94.6	0	.0
	Italy	0	.0	0	.0	0	.0	157	98.7
	Others	10	6.8	7	2.8	2	1.2	2	1.3
Gender	Woman	101	68.7	150	59.3	88	52.7	113	71.1
	Man	35	23.8	82	32.4	76	45.5	38	23.9
	Others	1	.7	1	.4	0	.0	0	.0
	Missing	10	6.8	20	7.9	3	1.8	8	5.0
	Elementary	0	0.0	2	.8	0	.0	11	6.9
Education	High school	45	30.6	50	19.8	30	18.0	57	35.8
	University	76	51.7	181	71.5	132	79.0	76	47.8
	Others	16	10.9	0	.0	2	1.2	8	5.0
	Missing	10	6.8	20	7.9	3	1.8	7	4.4
Sector	Extraction of raw materials	3	2.0	1	.4	3	1.8	0	.0
	Manufacturing	9	6.1	18	7.1	15	9.0	4	2.5
	Service to customers	37	25.2	92	36.4	68	40.7	15	9.4
	Public sector	35	23.8	27	10.7	11	6.6	91	57.2
	Non-government non-profit	3	2.0	7	2.8	6	3.6	1	.6
	Healthcare	5	3.4	9	3.6	8	4.8	7	4.4
	Education	34	23.1	36	14.2	30	18.0	19	11.9
	Others	11	7.5	41	16.2	22	13.2	12	7.5
	Missing	10	6.8	22	8.7	4	2.4	10	6.3
Leadership responsibility	No leadership responsibility	106	72.1	170	67.2	102	61.1	97	61.0
	Leadership responsibility	22	15.0	55	21.7	45	26.9	30	18.9
	Owner	0	.0	2	.8	7	4.2	4	2.5
	Others	8	5.4	4	1.6	9	5.4	19	11.9
	Missing	11	7.5	22	8.7	4	2.4	9	5.7
Full-time contract	Full-time	75	51.0	180	71.1	133	79.6	127	79.9
	Part-time	58	39.5	44	17.4	27	16.2	23	14.5
	Others	4	2.7	7	2.8	1	.6	150	94.3
	Missing	10	6.8	22	8.7	6	3.6	159	100.0
Pernament contract	Permanent	95	64.6	161	63.6	148	88.6	133	83.6
	Non-permanent	41	27.9	69	27.3	12	7.2	15	9.4
	Others	1	.7	1	.4	3	1.8	2	1.3
	Missing	10	6.8	22	8.7	4	2.4	9	5.7
White/blue collar	Blue-collar	3	2.0	5	2.0	5	3.0	8	5.0
	White-collar	111	75.5	203	80.2	133	79.6	94	59.1
	Balanced	23	15.6	23	9.1	25	15.0	45	28.3
	Missing	10	6.8	22	8.7	4	2.4	12	7.5

In Germany, some federal states and their cities started to declare the state of disaster on March 16th. On March 22nd, the government and the federal states introduced restriction of contact and activities. On April 20th, the government presented a 10-point-plan for the national health system and a week later, on April 27th, the obligations to wear a mask or other safety devices begun. Between April 30th and May 6th, the gradual easing of the restriction for public activities had begun. Due to new infections in some areas between the end of May and the beginning of June, the responsible federal states decided to reinstate restrictions on public activities (German Federal Ministry of Health, https://www.bundesgesundheitsministerium.de/coronavirus/chronik-coronavirus.html).

In Italy, the government declared the state of emergency on January 31st. The first public-activity restrictions (phase 1) were instated on February 23rd and since February 25th the government had been introducing new restrictions. On May 16th, the government launched the so-called phase 2 (May 18th – June 14th), restoring some commercial and public activities with the obligation of the use of safety devices. On June 11th, the government announced phase 3 (June 15th – July 14th) which still loosens – but does not remove – containment measures (Government office of the Italy, http://www.salute.gov.it/).

The lockdown and various security measures may have had serious consequences for employees. Some could not work or had to work from home and lose social (work) contact. Some employees lost part of their income due to the employer's problems or because their employer had no work for them. For other employees, the work has become more demanding and difficult due to the need to comply with safety measures or due to an increase in the workload (e.g. paramedics). Some employees took a risk of being infected with COVID-19 during their workday (e.g. cashiers, bus drivers). However, the means to counter the worst effects differed considerably across nations (e.g. short-time work, financial support). Our data describe the consequences of the pandemic and the security measures on the work conditions of employees in different European countries and the attitudes, perceived performance and resources of employees in the time of a pandemic. We also examined personal and organizational factors that could mitigate the potential negative impact of a pandemic situation.

The data were obtained in Germany, the Czech Republic, Slovakia and Italy during May 2020 and at the beginning of June 2020, when most security measures were still in place and employees had at least one month of experience with working under security measures.

**Table 3:** Work-related impact of pandemic.

		ΔWorkload	ΔWork difficulty	Income decrease	Risk of COVID-19	Social contact before pandemic	Social contact during pandemic	Home-office before pandemic	Home-office during pandemic
Germany	Valid	147	147	147	147	147	147	147	147
	Missing	0	0	0	0	0	0	0	0
	*M*	5.71	4.63	.22	2.78	5.57	1.86	1.36	7.41
	*SD*	2.39	3.17	.68	3.05	3.77	2.71	2.12	3.80
	Med	5	5	0	1	5	1	0	10
	Skew.	−0.05	−0.07	3.92	.85	−0.15	1.86	2.17	−1.15
	Kurt.	−0.38	−1.25	19.00	−0.52	−1.52	2.62	5.29	−0.38
	Min	0	0	0	0	0	0	0	0
	Max	10	10	5	10	10	10	10	10
Czech Republic	Valid	253	253	253	253	253	253	253	253
	Missing	0	0	0	0	0	0	0	0
	*M*	5.49	4.45	1.08	2.74	7.57	2.90	1.55	6.85
	*SD*	2.31	3.00	2.04	2.82	3.27	3.26	2.17	3.87
	Med	5	5	0	2	10	2	1	9
	Skew.	.10	.05	2.35	.98	−1.09	1.02	1.81	−0.85
	Kurt.	−0.56	−1.01	5.33	.01	−0.24	−0.27	2.90	−0.91
	Min	0	0	0	0	0	0	0	0
	Max	10	10	10	10	10	10	10	10
Slovakia	Valid	167	167	167	167	167	167	167	167
	Missing	0	0	0	0	0	0	0	0
	*M*	5.75	4.84	1.38	2.74	6.96	2.81	1.66	6.81
	*SD*	2.43	3.01	2.09	3.06	3.53	3.53	2.28	4.08
	Med	5	6	0	2	8	1	1	9
	Skew.	−0.08	−0.30	2.06	.96	−0.79	1.08	2.03	−0.81
	Kurt.	−0.46	−1.14	4.74	−0.21	−0.81	−0.29	4.42	−1.11
	Min	0	0	0	0	0	0	0	0
	Max	10	10	10	10	10	10	10	10
Italy	Valid	159	159	159	159	159	159	159	159
	Missing	0	0	0	0	0	0	0	0
	*M*	6.28	6.99	1.35	3.61	8.15	2.04	.95	7.46
	*SD*	2.34	2.94	2.49	3.28	2.61	2.82	2.24	3.58
	Med	6	8	0	3	10	1	0	10
	Skew.	−0.11	−1.14	2.38	.47	−1.34	1.56	2.79	−1.23
	Kurt.	−0.71	.44	5.18	−0.90	.86	1.46	7.37	.01
	Min	0	0	0	0	0	0	0	0
	Max	10	10	10	10	10	10	10	10
Full sample	Valid	726	726	726	726	726	726	726	726
	Missing	0	0	0	0	0	0	0	0
	*M*	5.77	5.13	1.03	2.94	7.15	2.48	1.40	7.09
	*SD*	2.38	3.18	2.02	3.04	3.42	3.16	2.21	3.85
	Med	5	6	0	2	9	1	0	9
	Skew.	−0.02	−0.24	2.62	.82	−0.86	1.28	2.10	−0.97
	Kurt.	−0.56	−1.13	7.16	−0.44	−0.69	.38	4.36	−0.72
	Min	0	0	0	0	0	0	0	0
	Max	10	10	10	10	10	10	10	10

**Table 4:** Attitudes, feelings and performance of employees.

		Job satistfaction	Intention to leave	Work intensification	Actual task performance	ΔTask performance	Actual org. commitment	ΔOrg. commitment	Irritation - cognitive	Irritation - affective
Germany	Valid	137	137	147	147	147	147	147	147	147
	Missing	10	10	0	0	0	0	0	0	0
	*M*	7.16	3.81	2.55	3.58	3.04	3.78	3.04	2.73	2.43
	*SD*	1.93	3.64	1.08	.93	.86	.88	.61	1.09	1.07
	Med	7	2	2.50	3.60	3.00	4.00	3.00	2.67	2.33
	Skew.	−0.96	.54	.48	−0.43	.03	−1.02	.03	.42	.48
	Kurt.	.99	−1.22	−0.42	−0.57	1.06	1.06	3.42	−0.83	−0.57
	Min	1	0	1	1	0	1	1	1	1
	Max	10	10	5	5	5	5	5	5	5
Czech Republic	Valid	231	230	253	253	253	252	252	253	253
	Missing	22	23	0	0	0	1	1	0	0
	*M*	7.19	4.95	2.41	3.80	3.10	3.57	3.11	2.74	2.44
	*SD*	2.08	3.56	1.07	.88	.77	.83	.70	1.03	1.00
	Med	8	5	2.25	4.00	3.00	3.75	3.00	2.67	2.33
	Skew.	−1.13	.11	.28	−0.68	.51	−0.81	.29	.21	.37
	Kurt.	1.05	−1.40	−0.96	.06	.78	1.02	1.99	−0.85	−0.65
	Min	0	0	1	1	1	1	1	1	1
	Max	10	10	5	5	5	5	5	5	5
Slovakia	Valid	163	163	167	167	166	167	166	167	167
	Missing	4	4	0	0	1	0	1	0	0
	*M*	7.20	4.01	2.53	4.04	3.24	3.65	3.12	2.78	2.53
	*SD*	2.33	3.38	1.21	.80	.77	.87	.92	1.05	1.07
	Med	8	3	2.50	4.20	3.00	4.00	3.00	2.67	2.33
	Skew.	−1.19	.46	.35	−0.80	.36	−0.86	−0.14	.09	.34
	Kurt.	1.44	−1.15	−0.90	−0.01	.49	.76	.70	−0.95	−0.92
	Min	0	0	1	2	1	1	1	1	1
	Max	10	10	5	5	5	5	5	5	5
Italy	Valid	150	148	159	159	159	158	158	159	158
	Missing	9	11	0	0	0	1	1	0	1
	*M*	7.60	2.73	2.62	3.67	3.20	4.32	3.46	2.90	2.32
	*SD*	1.68	3.46	1.00	.93	.91	.66	.78	1.00	1.06
	Med	8	1	2.50	3.80	3.00	4.25	3.00	3.00	2.00
	Skew.	−1.06	.97	.32	−0.41	.14	−1.57	.25	−0.37	.42
	Kurt.	1.98	−0.54	−0.67	−0.40	−0.02	4.53	.80	−0.58	−0.82
	Min	1	0	1	1	1	1	1	1	1
	Max	10	10	5	5	5	5	5	5	5
Full sample	Valid	681	678	726	726	725	724	723	726	725
	Missing	45	48	0	0	1	2	3	0	1
	*M*	7.28	4.01	2.51	3.78	3.14	3.79	3.17	2.79	2.43
	*SD*	2.04	3.60	1.09	.90	.82	.86	.77	1.04	1.04
	Med	8	3	2.50	4.00	3.00	4.00	3.00	2.67	2.33
	Skew.	−1.16	.43	.34	−0.60	.26	−0.92	.13	.11	.39
	Kurt.	1.49	−1.27	−0.75	−0.24	.57	.98	1.49	−0.88	−0.74
	Min	0	0	1	1	0	1	1	1	1
	Max	10	10	5	5	5	5	5	5	5

In total, 1.372 respondents started our survey. We excluded some of them from the sample according to the pre-set conditions. 552 respondents did not answer 30% or more questions related to the research variables. Another 22 respondents were employed in their organization for less than 5 months and therefore could not assess the changes associated with the pandemic. 72 respondents did not work in Germany, the Czech Republic, Slovakia and Italy. Therefore, the presented dataset consists of responses of 726 people who were employed in Germany, the Czech Republic, Slovakia or Italy during the COVID-19 pandemic. The socio-demographic and job-related characteristics of the sample are described in Table 1 and Table 2.

Table 3 describes how the pandemic and lockdown affected the work of employees in each of the 4 countries. Table 4 describes the attitudes, feelings and perceived performance of employees in the time of the pandemic and also the perceived change in their performance and organizational commitment in comparison to the time before pandemic. Table 5 describes organization-related (servant leadership of the supervisor, perceived organizational support, social support from colleagues) and personal (resilience, occupational self-efficacy, job crafting, readiness for change) characteristics that might help employees cope with the pandemic situation and lockdown. The variables that were measured by scales with several items (see Table 7) were computed as a mean of all valid answers provided by each respondent. The McDonald's omegas which indicate the internal consistency of the scales are presented in Table 7. Code book and all variables are available in the associated dataset (http://dx.doi.org/10.17632/77dcsp2vcw.2) in raw form. The dataset enables describing and analysing the work-relates consequences of COVID-19 pandemic in various countries and examining the moderation effect of organizational and personal factors.

**Table 5:** Organizational and personal factors.

		Servant leadership	Perceived organizational support	Resilience	Social support	Occupational self-efficacy	Job crafting - resources	Job crafting - demands	Readiness for change
Germany	Valid	146	146	141	146	141	139	139	139
	Missing	1	1	6	1	6	8	8	8
	*M*	4.04	3.06	3.81	4.14	3.87	2.62	2.57	3.64
	*SD*	1.35	1.02	.56	.86	.75	1.05	.72	.77
	Med	4.36	3.17	3.80	4.33	4.00	2.67	2.50	3.67
	Skew.	−0.76	−0.17	−0.25	−1.36	−0.54	.06	.28	−0.28
	Kurt.	.42	−0.66	.05	2.23	.68	−0.45	−0.24	.16
	Min	0	1	2	1	1	0	1	1
	Max	7	5	5	5	5	5	5	5
Czech Republic	Valid	247	253	233	253	233	231	233	232
	Missing	6	0	20	0	20	22	20	21
	*M*	4.25	3.21	3.55	4.18	3.77	2.75	2.96	3.61
	*SD*	1.11	.96	.66	.76	.71	1.03	.77	.85
	Med	4.43	3.33	3.60	4.33	4.00	2.67	3.00	4.00
	Skew.	−0.37	−0.37	−0.56	−1.04	−0.73	.05	−0.21	−0.76
	Kurt.	−0.31	−0.18	.81	1.17	.99	−0.78	−0.11	.68
	Min	1	1	1	2	1	1	1	1
	Max	7	5	5	5	5	5	5	5
Slovakia	Valid	161	165	164	166	164	162	163	164
	Missing	6	2	3	1	3	5	4	3
	*M*	4.07	3.12	3.61	4.02	3.81	2.44	3.08	3.50
	*SD*	1.17	1.08	.62	.90	.80	.97	.77	.85
	Med	4.00	3.00	3.60	4.00	4.00	2.33	3.17	3.67
	Skew.	−0.09	−0.26	−0.27	−0.79	−0.78	.47	−0.25	−0.46
	Kurt.	−0.55	−0.65	.58	.18	.80	−0.21	.47	.40
	Min	1	1	1	1	1	1	1	1
	Max	7	5	5	5	5	5	5	5
Italy	Valid	152	156	156	156	156	154	156	155
	Missing	7	3	3	3	3	5	3	4
	*M*	4.31	3.27	3.57	3.97	3.74	2.89	2.65	3.55
	*SD*	1.16	.94	.74	.94	.57	.93	.74	.79
	Med	4.43	3.33	3.55	4.00	3.67	3.00	2.67	3.67
	Skew.	−0.42	−0.26	−0.37	−0.78	−0.27	−0.25	.26	−0.04
	Kurt.	−0.28	−0.36	−0.01	−0.17	.52	−0.39	−0.08	−0.07
	Min	1	1	1	1	2	1	1	1
	Max	7	5	5	5	5	5	5	5
Full sample	Valid	706	720	694	721	694	686	691	690
	Missing	20	6	32	5	32	40	35	36
	*M*	4.18	3.17	3.62	4.09	3.79	2.68	2.84	3.58
	*SD*	1.19	1.00	.66	.86	.71	1.01	.78	.82
	Med	4.29	3.33	3.60	4.33	4.00	2.67	2.83	3.67
	Skew.	−0.46	−0.29	−0.45	−1.00	−0.62	.07	.00	−0.46
	Kurt.	−0.01	−0.44	.53	.76	.89	−0.62	−0.23	.36
	Min	0	1	1	1	1	0	1	1
	Max	7	5	5	5	5	5	5	5

## 2 Design, materials and methods

We obtained the data via an online survey. The survey was promoted at social networks, in articles in online newspapers, by direct emails and in a university newsletter. We formulated new items to measure the impact of pandemic and lockdown on the work of employees and to measure job satisfaction and intention to leave (see Table 6). To measure organizational commitment, work performance, irritation, work intensification and various organizational and personal factors, we used established questionnaires (see Table 7). We modified the instructions and response scale (see Appendix) of Individual Work Performance Questionnaire and Klein's unidimensional scale of commitment to be able to measure the change in task performance and organizational commitment during the pandemic. We also modified the instructions of Irritation scale and Work intensification scale to measure irritation and work intensification in time of the pandemic and lockdown (see Appendix).

**Table 6:** Items used to describe work-related consequences of COVID-19, job satisfaction and intention to leave.

Variable label	Variable	Item	Response scale
CWorkl	Change in workload	How has actual pandemic situation changed your workload?	0 (I have much less work to do) - 10 (I have much more work to do)
WDiffic	Increased work difficulty	How much has the pandemic situation increased the difficulty of your work (eg because of the need to wear protective equipment, because of increased hygiene, because of the need to communicate online)?	0 (not at all) - 10 (very much)
Income	Decreased income	How much has your monthly income decreased as a result of the pandemic situation and lockdown?	0 (it is the same or higher) - 10 (I completely lost my income)
COVrisk	Risk of COVID-19	How big is the risk being infected with COVID-19 during your work?	0 (no risk) - 10 (very high risk)
SocPast	Social contact before pandemic	How often have you personally met other people (colleagues, customers, suppliers) during the workday before the pandemic and lockdown?	0 (not at all) - 10 (all the time)
SocNow	Social contact during pandemic	How often do you personally meet other people (colleagues, customers, suppliers) during the pandemic and lockdown?	0 (not at all) - 10 (all the time)
HOpast	Home-office before pandemic	How often did you work from home before the pandemic and lockdown?	0 (not at all) - 10 (all the time)
HOnow	Home-office during pandemic	How often do you work from home now in the time of the pandemic and lockdown?	0 (not at all) - 10 (all the time)
Satisf	Job satisfaction	Are you generally satisfied with your current job?	0 (not at all) - 10 (very much)
IntLeave	Intention to leave	Do you want to leave your job and your organization in following 3 years?	0 (certainly not) - 10 (certainly yes)

**Table 7:** List of questionnaires and internal consistency of each scale.

			Number	Response	McDonald's ω		
Label	Variable	Source	of items	scale	GE	CZ	IT
WIntens	Work intensification	Intensified Job Demands Scale [10], modified instructions	4	1–5	.842	.822	.728
APerf	Actual task performance	Individual Work Performance Questionnaire, Task performance subscale [11]	5	1–5	.892	.869	.872
CPerf	Change in task performance	Individual Work Performance Questionnaire, Task performance subscale, [11], modified instructions	5	1–5	.914	.894	.912
ACommit	Actual organizational commitment	Klein et al.’s Unidimensional Target-free Scale of Commitment (target = organization) [12]	4	1–5	.895	.883	.835
CCommit	Change in organizational commitment	Klein et al.’s Unidimensional Target-free Scale of Commitment (target = organization) [12], modified instructions	4	1–5	.881	.920	.893
IritC	Irritation - cognitive	Irritation Scale, Cognitive subscale [7]	3	1–5	.870	.786	.852
IritA	Irritation - affective	Irritation Scale, Affective subscale [7]	3	1–5	.921	.849	.912
Servant	Servant leadership	Servant Leadership Questionnaire, SL-7 [13]	7	1–7	.876	.811	.872
POS	Perceived organizational support	Survey of Perceived Organizational Support – short 3 item version [14]	3	1–5	.830	.860	.880
Resil	Resilience	Short version of the Connor-Davidson Resilience Scale CD-RISC-10 [15]	10	1–5	.840	.867	.896
SocSup	Social support	Social Support Scale [16]	3	1–5	.878	.874	.876
OcSEff	Occupational self-efficacy	Occupational Self-efficacy Scale – short [17]	3	1–5	.809	.805	.704
JCraftR	Job crafting - resources	Job Crafting Scale, Increasing job resources subscale [18]	3	1–5	.805	.775	.768
JCraftD	Job crafting - demands	Job crafting scale, Hindering job demands subscale [18]	6	1–5	.744	.739	.813
ReadCh	Readiness for change	Organizational Change Questionnaire [19]	3	1–5	.874	.896	.880

Note. McDonald's omegas are provided for three language versions of questionnaires; German (GE): *N* = 141, Czech (CZ): *N* = 366; Italian (IT): *N* = 158; Slovak respondents filled out mostly the Czech version; 61 respondents filled out the English questionnaire.

The survey was available in English, German, Czech (for Czech and Slovak respondents) and Italian. We used official and published translations of each questionnaire if it was available. If there was no official translation, we did two to three independent translations from English and then a backtranslation to English to ensure the quality of the new language adaptation.

In the German survey, we used the original German scales (see Table 7) for measuring irritation, occupational self-efficacy, social support and work intensification. We also used official translations of Job Crafting Scale [1], Klein's Unidimensional Scale of Commitment (translated by Vitera, https://u.osu.edu/commitmentmeasure/k-u-t-commitment-measure/german/), Survey of Perceived Organizational Support (translated by Siebenaler & Fischer, https://doi.org/10.6102/zis277), Readiness for Change Scale (presented by Scheel at the 33rd Annual SIOP Conference, 2018) and Connor-Davidson Resilience Scale (translated by Krähenmann & Krausenick, www.connordavidson-resiliencescale.com). Scales for measuring work performance and servant leadership were newly translated by an author of this article and a class of Master students.

In the Czech survey (for Czech and Slovak respondents), we used published translation of Klein's Unidimensional Scale of Commitment [2] and Servant Leadership Questionnaire [3], official unpublished translation of Connor-Davidson Resilience Scale (translated by Dostalova et al., www.connordavidson-resiliencescale.com) and existing unpublished translations of Individual Work Performance Questionnaire (psychometric characteristics available in [4]) and Survey of Perceived Organizational Support (translated by Stejdirova et al., https://is.muni.cz/th/gkk6r/Stejdirova_bakalarska_prace.pdf). Irritation Scale, Occupational Self-efficacy Scale, Social Support Scale, Intensified Job Demands Scale, Job Crafting Scale and Organizational Change Questionnaire were newly translated by authors of this article.

In the Italian survey, we used published translation of Work Performance Questionnaire [5], Klein's Unidimensional Scale of Commitment [6], Irritation Scale [7], Survey of Perceived Organizational Support [8], subscale “Increasing job resources" from Job Crafting Scale [9] and the official translation of Connor-Davidson Resilience Scale (translated by Comoretto, www.connordavidson-resiliencescale.com). The Occupational Self-efficacy Scale, Servant Leadership Questionnaire, Social Support Scale, Intensified Job Demands Scale, Organizational Change Questionnaire and subscale "Hindering job demands" from Job Crafting Scale were newly translated by authors of this article.

## Ethics statement

Informed consent was obtained from all respondents before they started the survey. They were informed that the survey was anonymous and that they could stop at any time.

## Declaration of Competing Interest

The authors declare that they have no known competing financial interests or personal relationships which have, or could be perceived to have, influenced the work reported in this article.

## Appendix – modified instructions and response scales

### Modified instructions for Individual Work Performance Questionnaire for measuring change in task performance and for Klein's unidimensional scale of commitment for measuring change in organizational commitment

*Now use the same items to compare your performance and attitudes in the past month during the pandemic and lockdown to your performance and attitudes in 3 months before the pandemic situation and lockdown occurred.*

*The answer "significantly less often" / "much less" means that your performance/attitude is lower/weaker now than it was before the pandemic.*

*Now, in the time of pandemic and lockdown (in comparison to the situation before the pandemic and lockdown)…*

### Modified response scale for Individual Work Performance Questionnaire for measuring change in task performance

1 = significantly less often, 2 = slightly less often, 3 = similarly often, 4 = slightly more often, 5 = significantly more often

### Modified response scale for Klein's unidimensional scale of commitment for measuring change in organizational commitment

*1 = much less, 2 = slightly less, 3 = similarly, 4 = slightly more, 5 = much more*

### Modified instructions for Irritation questionnaire

*Please indicate how much you agree with the following statements.*

*In the past month, during the pandemic situation:*

### Modified instructions for Work intensification questionnaire

*In the past month, during the pandemic and lockdown…*

## Appendix B Supplementary materials

Image, application 1

Image, application 2
